# BORIS/CTCFL promotes a switch from a proliferative towards an invasive phenotype in melanoma cells

**DOI:** 10.1038/s41420-019-0235-x

**Published:** 2020-01-02

**Authors:** Sanne Marlijn Janssen, Roy Moscona, Mounib Elchebly, Andreas Ioannis Papadakis, Margaret Redpath, Hangjun Wang, Eitan Rubin, Léon Cornelis van Kempen, Alan Spatz

**Affiliations:** 1 Lady Davis Institute for Medical Research, Montréal, QC Canada; 2 Department of Pathology, McGill University, Montréal, QC Canada; 3 The Shraga Segal Department of Microbiology, Immunology and Genetics, Ben-Gurion University of the Negev, Beer Sheva, Israel; 4 Division of Pathology, Department of Laboratory medicine, McGill University Health Center, Montreal, QC Canada; 5 Department of Pathology, Laboratory for Molecular Pathology, University Medical Center Groningen, Groningen, The Netherlands; 6 Department of Oncology, McGill University, Montréal, QC Canada

**Keywords:** Melanoma, Cell growth

## Abstract

Melanoma is among the most aggressive cancers due to its tendency to metastasize early. Phenotype switching between a proliferative and an invasive state has been suggested as a critical process for metastasis, though the mechanisms that regulate state transitions are complex and remain poorly understood. Brother of Regulator of Imprinted Sites (BORIS), also known as CCCTC binding factor-Like (CTCFL), is a transcriptional modulator that becomes aberrantly expressed in melanoma. Yet, the role of BORIS in melanoma remains elusive. Here, we show that BORIS is involved in melanoma phenotype switching. Genetic modification of BORIS expression in melanoma cells combined with whole-transcriptome analysis indicated that BORIS expression contributes to an invasion-associated transcriptome. In line with these findings, inducible BORIS overexpression in melanoma cells reduced proliferation and increased migration and invasion, demonstrating that the transcriptional switch is accompanied by a phenotypic switch. Mechanistically, we reveal that BORIS binds near the promoter of transforming growth factor-beta 1 (*TFGB1*), a well-recognized factor involved in the transition towards an invasive state, which coincided with increased expression of *TGFB1*. Overall, our study indicates a pro-invasive role for BORIS in melanoma via transcriptional reprogramming.

## Introduction

Primary cutaneous melanoma is one of the most aggressive cancers due to its ability to rapidly disseminate and develop distant metastases. During melanoma progression, cells undergo a reversible transition from a proliferative to an invasive state, a process known as “phenotype switching”, which closely resembles epithelial-mesenchymal transition (EMT)^1,2^. Gene expression profiling of melanoma cell lines and tumor samples has revealed the different transcriptional states that underlie the proliferative and invasive phenotypes of melanoma cells^3–7^. While high expression of the transcription factor MITF is linked to the proliferative state, TGF-beta was the first factor associated with expression of invasion-associated genes^3^. Currently, various other transcriptional regulators have been implicated in the switch towards an invasive state, including BRN2, AP-1, TEAD, NFATC2, and NFIB^4,8–11^. Furthermore, integration of transcriptomic and epigenomic data from melanoma cells revealed widespread differences in the chromatin landscape between the proliferative and invasive cellular states^4^. While these findings have greatly contributed to our understanding of phenotype switching, new insights may be derived from the identification of factors that regulate the transcriptional landscape either directly as transcriptional regulator or indirectly by changing the chromatin landscape

Brother of Regulator of Imprinted Sites (BORIS) is a DNA-binding protein with high similarity to CCCTC binding factor (CTCF)^12^, a multifunctional transcription factor that plays an important role in chromatin organization^13^. In contrast to CTCF’s tumor suppressive functions^14,15^, multiple studies have indicated an oncogenic role for BORIS^16–20^. Like CTCF, BORIS plays a role in transcriptional regulation^17,18,21,22^. The mechanisms through which BORIS can alter transcription rely on BORIS’ ability to bind the DNA at specific binding motifs^23–25^. BORIS can alter transcription by acting as a transcriptional activator^17,18,22,26^, recruiting a transcriptional activator^27^, altering DNA methylation^17,28,29^ and histone modifications^17,21,22,28^, or recruiting chromatin-modifying proteins^30,31^. Furthermore, it is believed that BORIS impacts the chromatin landscape by interfering with CTCF-mediated chromatin loops^24,32^.

BORIS expression is normally restricted to the testis and becomes aberrantly expressed in different types of cancer, hence the designation of BORIS as a cancer testis antigen^12^. In melanoma, *BORIS* expression was observed in 59% of melanoma cell lines, in 16% of primary melanomas and in 34% of melanoma metastases, with *BORIS* reaching similar expression levels as observed in the testis^33^. Importantly, no *BORIS* expression was observed in normal human skin tissue^33^. While these observations suggest that BORIS can play a role in melanoma progression, little is known about BORIS functions in melanoma development and progression.

Herein we sought to determine if BORIS plays a role in melanoma progression through its function as transcriptional modulator. Using a doxycycline-inducible expression system in melanoma cells we found that BORIS expression led to upregulation of genes that were enriched among invasion-related processes and gene signatures, while downregulated genes were enriched among proliferation-related processes and gene signatures. Accordingly, we observed reduced proliferation and increased migratory and invasive abilities of melanoma cells upon BORIS expression. Furthermore, we found that BORIS binds near the *TGFB1* promoter, which co-occurs with increased expression of *TGFB1* and its target genes. These findings identify BORIS as a mediator of transcriptional reprogramming in melanoma cells, resulting in a switch towards an invasive phenotype.

## Results

### Aberrant *BORIS* expression in melanoma

To corroborate previous findings that demonstrated *BORIS* expression in human melanoma tumors, but not in normal human skin^33^, we analyzed *BORIS* expression data for testis, skin, and melanoma samples and found significantly higher *BORIS* expression in melanoma and testis compared to skin samples (Fig. S1A). In addition, *BORIS* expression among metastatic melanoma samples was significantly higher compared to primary melanoma samples (Fig. S1B). Furthermore, in our panel of melanoma and non-malignant congenital nevi cell lines we observed *BORIS* in melanoma cell lines, but not in the congenital nevi cells (Figure S1C). Collectively, these data confirm that BORIS is aberrantly expressed in melanoma cell lines and tumor samples compared to non-malignant cell lines and normal skin samples, suggesting that BORIS may play an important role in melanoma development and metastasis.

### BORIS expression results in reduced proliferation and increased apoptosis

**Fig. 1:**
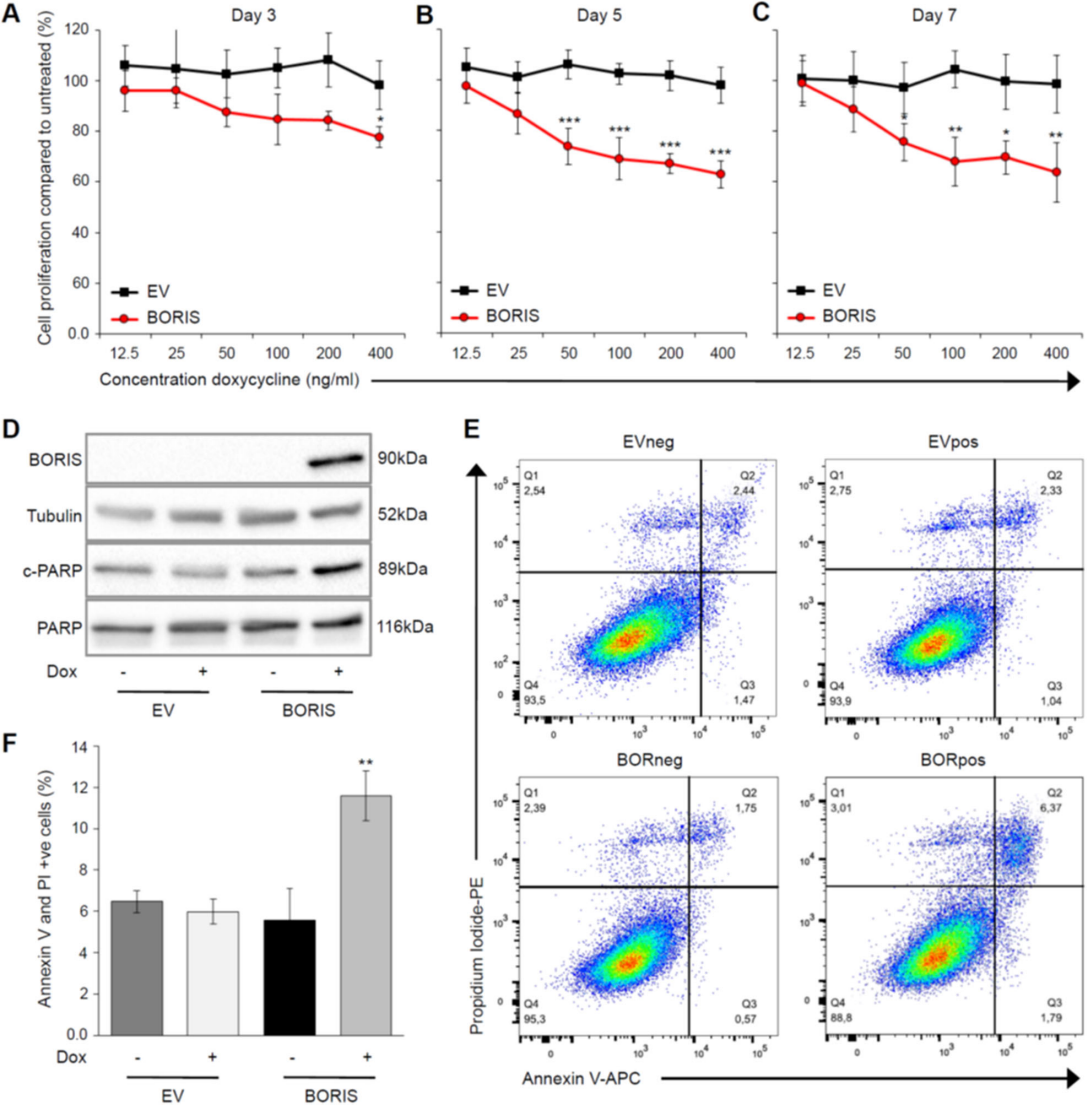
BORIS expression in melanoma cells results in reduced proliferation and increased apoptosis. **a–c** Cell proliferation upon expression of BORIS protein in the MM057 cell line with the indicated concentration of dox for 3, 5, or 7 days of cell culture. Two independent experiments were performed in technical triplicate. **d–f** BORIS expression was induced in MM057 cells with 50 ng/ml dox for 5 days. **d** Whole-cell lysate was used for immunoblotting (*n* = 3) with anti-BORIS, anti-cleaved PARP (c-PARP) and anti-PARP antibodies. Anti-Tubulin was used as a loading control. **e** Bar graph representing the mean percentage of Annexin V- and PI-positive cells as determined by flow cytometry (*n* = 3). **f** Representative image of the percentage of apoptotic and necrotic cells as determined by flow cytometry analysis of Annexin V and PI staining. Error bars represent the standard error of mean. Asterisk indicates significantly different from **A-** untreated, **E-** the control (*P < 0.05. **P < 0.01, ***P < 0.001). Dox doxycycline.

To gain insight into the role of BORIS in melanoma, we first established a doxycycline (dox)-inducible model of BORIS expression in the MM057 melanoma cell line, which expresses low *BORIS* mRNA compared to other melanoma cell lines (Fig. S1C). The expression of BORIS in the presence of dox was confirmed by immunoblot (Fig. S2A). A striking reduction in cell number was observed during cell culture of BORIS-expressing cells [BORIS with dox (BORpos)] compared to the control cells [empty vector without dox (EVneg) and with dox (EVpos), and BORIS without dox (BORneg)] (Fig. S2B). We used this phenotype to optimize the level of BORIS expression (dox concentration and duration) for further characterization of BORIS’ role in melanoma cells. To this end, BORIS expression was induced with increasing concentration of dox for 3, 5, or 7 days. Expression of BORIS for 3 days did not significantly reduce cell proliferation compared to untreated cells, except in the presence of the highest concentration of dox (Fig. 1a). After 5 and 7 days of BORIS expression we observed a significant reduction in proliferation even at low dox concentrations, but not for EV cells, indicating that dox itself does not affect proliferation (Fig. 1b, c). Immunoblot analysis confirmed expression of BORIS at each time point. (Fig. S2C). Based on these results, BORIS expression was induced with 50–100 ng/ml dox for five days to further dissect BORIS functions in melanoma.

As altered BORIS expression has an effect on apoptosis in breast and colon cancer cell lines^16,34^, we addressed whether the observed reduction in proliferation is due to BORIS-induced apoptosis. Compared to control cells, BORpos cells demonstrated enhanced expression of cleaved PARP, a marker for cell death (Fig. 1d). In addition, we observed increased early apoptotic, apoptotic and necrotic cells following BORIS expression compared to controls (Fig. 1e, f), confirming that BORIS expression in melanoma cells leads to a slight, though significant, increase in apoptosis. Collectively, our results show that expression of BORIS in melanoma cells leads to decreased proliferative activity, which is in part due to apoptosis.

### BORIS expression induces large-scale differential gene expression

**Fig. 2:**
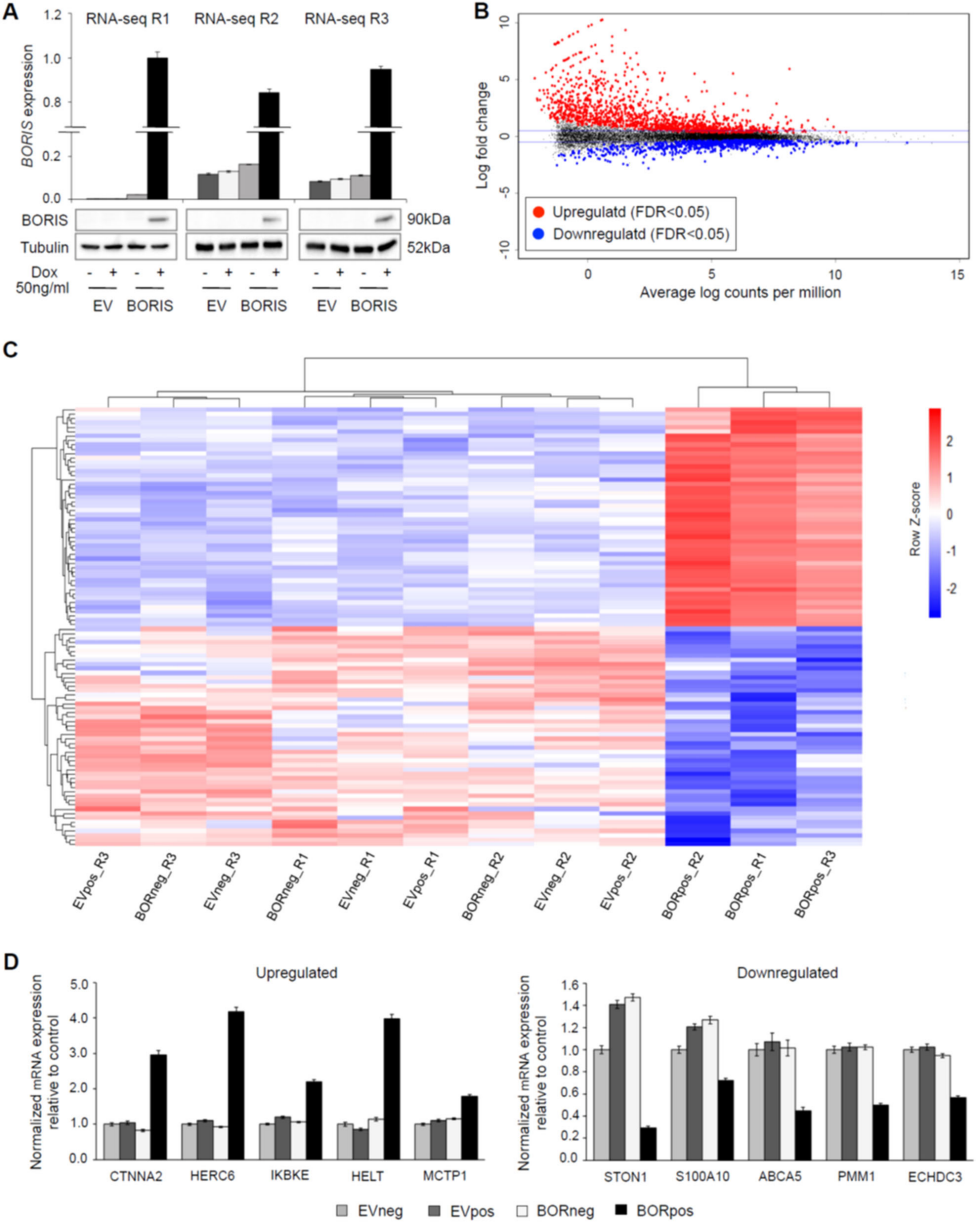
BORIS expression in melanoma cells leads to large-scale differential gene expression. **a**, **d** BORIS expression was induced in the MM057 cell line using 50 ng/ml dox for 5 days. **a** Expression of BORIS mRNA and protein as determined by qPCR and immunoblot. Anti-Tubulin was used as a loading control for immunoblot. **b** MA plot representing the RNA-seq data of all BORneg versus BORpos samples (*n* = 3). The blue horizontal lines mark log_2_ fold change = 0.5. **c** Heat map representing the log_2_ counts per million for the top 100 up and downregulated genes across all RNA-seq samples. **d** qPCR for upregulated and downregulated genes. For each qPCR experiment, a technical triplicate was performed for two biological replicates. Expression was normalized to *HPRT1* and *TBP*. Error bars represent the standard error of the mean. R replicate, dox doxycycline, FDR false discovery rate.

Given that BORIS plays a role in transcriptional regulation^17,18,21,22^, we investigated the effect of BORIS expression on the transcriptome of melanoma cells using RNA-seq. BORIS expression was induced in the MM057 cells and expression was confirmed at the mRNA and protein level (Fig. 2a). Unsupervised analysis of the RNA-seq data demonstrated clustering of the samples into two groups, one representing the controls and the other BORpos cells (Fig. S3). Differential gene expression analysis between BORneg and BORpos cells identified 2045 significant differentially expressed genes (DEGs): 1308 upregulated and 737 downregulated (Fig. 2b and Table S2). Analysis of the log2 counts per million for the top 100 up and downregulated genes demonstrated robust differential expression (Fig. 2c). To validate the RNA-seq dataset, BORIS expression was induced in the MM057 cells followed by qPCR for genes distributed throughout the lists of upregulated and downregulated DEGs (Fig. 2d). Together, these data demonstrate that ectopic BORIS expression in MM057 cells results in large-scale differential gene expression.

### BORIS expression contributes to a switch from a proliferative to invasive transcriptional state

**Fig. 3:**
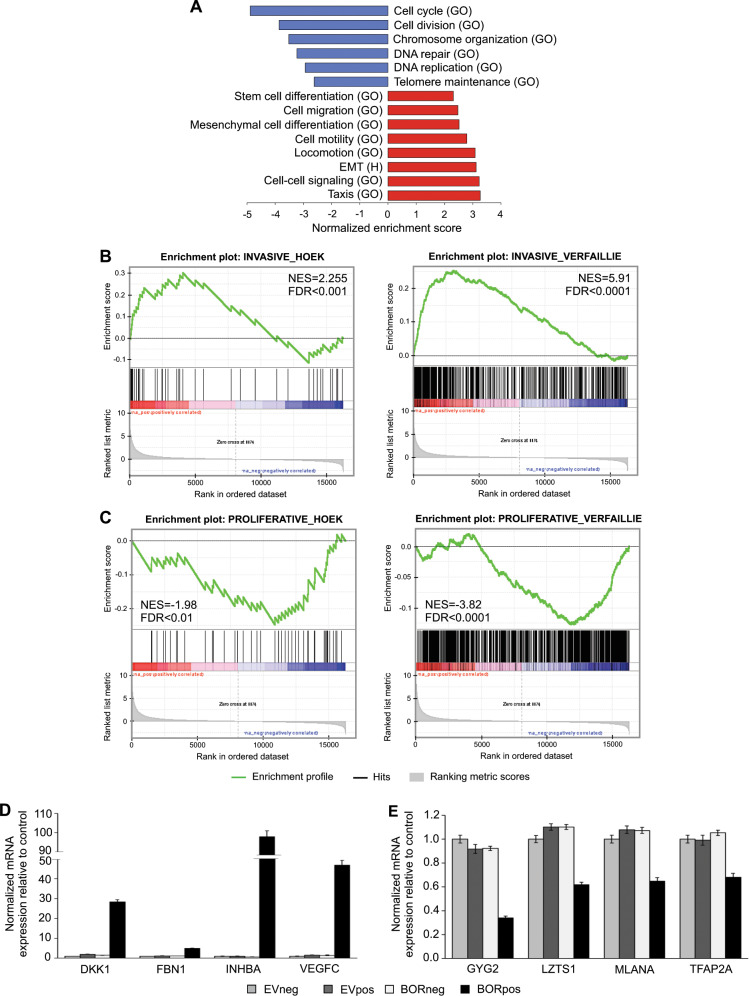
BORIS promotes a switch from a proliferative to invasive gene signature in melanoma cells. **a–c** GSEA on the RNA-seq data from the MM057 melanoma cell line for **a** biological processes and the hallmark EMT gene set from the Molecular Signatures Database, **b** the invasive, and **c** proliferative gene signatures. **d**, **e** BORIS expression was induced in the MM057 cell line using 50 ng/ml doxycycline for 5 days followed by qPCR for (**d**) invasive and (**e**) proliferative genes. For each sample, a technical triplicate was performed for two biological replicates. Expression was normalized to *HPRT1* and *TBP*. Error bars represent the standard error of mean. GO gene ontology (biological process), H Hallmark gene set, NES normalized enrichment score, FDR false discovery rate.

We used the RNA-seq dataset for gene set enrichment analysis (GSEA)^35^ to identify potential BORIS-mediated biological processes. In agreement with reduced proliferation (Fig. 1b, c), we observed a strong negative enrichment (FDR < 0.01) for proliferation-related processes (Fig. 3a). Interestingly, we found a strong positive enrichment (FDR < 0.01) for migration and invasion-related processes, including EMT, cell motility, and locomotion (Fig. 3a). Notably, the MM057 melanoma cell line that was used for the RNA-seq experiment harbors a proliferative transcriptome^4^. Together, these findings suggest that BORIS contributes to a switch from a proliferative to invasive transcriptome in melanoma cells.

To further explore if BORIS plays a role in phenotype switching we performed GSEA for the melanoma-specific proliferative and invasive Hoek^3^ and Verfaillie^4^ gene signatures. For these gene sets, we identified a strong positive correlation with the invasive gene signatures (Fig. 3b) and a negative correlation with the proliferative gene signatures (Fig. 3c). Furthermore, when we compared the upregulated DEGs from the RNA-seq data we observed a significant overlap between invasion-associated genes and the upregulated DEGs, but not between the proliferation-associated genes and the upregulated DEGs (Fig. S5A). A similar analysis with the downregulated DEGs revealed a significant overlap with proliferation-associated genes, but not with invasion-associated genes (Fig. S5B). These data indicate that BORIS enables an invasion-like transcriptional state.

To validate the effect of BORIS expression on the above-described signatures we induced BORIS expression in MM057 cells and performed qPCR for four upregulated and four downregulated DEGs that are part of both the Hoek and Verfaillie invasive and proliferative signatures, respectively. Our results demonstrated a significant upregulation of invasion-associated genes (Fig. 3d) and a significant downregulation of proliferation-associated genes (Fig. 3e) in the BORpos cells compared to the control cells. Together, these results indicate that BORIS promotes a switch from a proliferative towards an invasive transcriptional state in MM057 melanoma cells.

### BORIS expression promotes an invasive phenotype

**Fig. 4:**
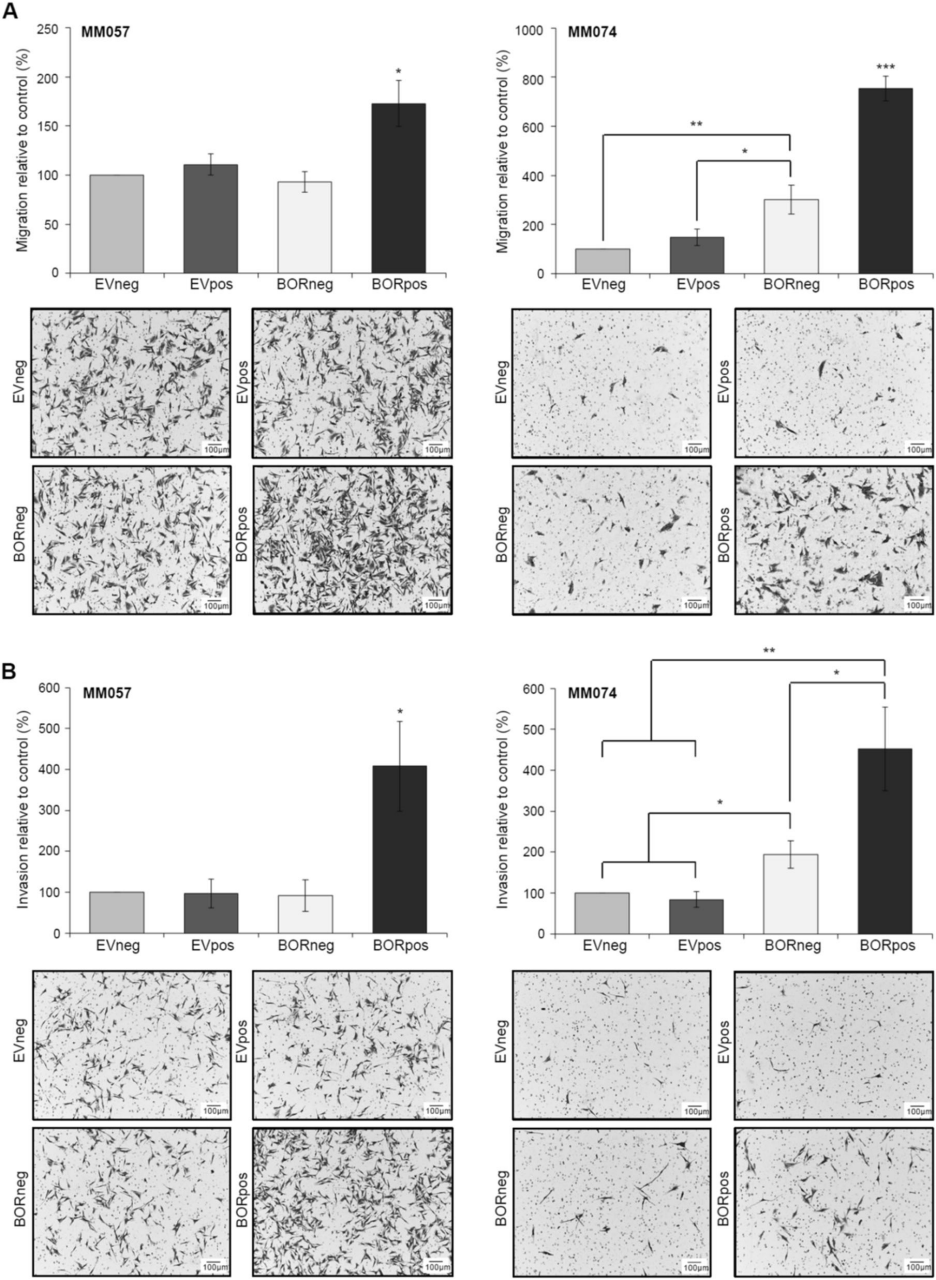
BORIS expression results in increased migration and invasion of melanoma cells with a proliferative gene signature. **a**, **b** BORIS expression was induced in the MM057 and MM074 melanoma cell lines using 100 ng/ml doxycycline for 5 days. In vitro cell **a** migration and **b** invasion was determined by transwell assays. Bar graphs display the percentage of cells per field that **a** migrated and **b** invaded through the membrane and are accompanied by a representative image. For each assay, images of six fields were captured and counted using the Cell Counter option in ImageJ. Three independent experiments were performed, each consisting of two technical replicates. Asterisk indicates significantly different from the controls (*P < 0.05. **P < 0.01, ***P < 0.001).

Next, we set out to determine if BORIS expression promotes an invasive phenotype in melanoma cells. To address this, we first assessed the effect of ectopic BORIS expression on the migration of melanoma cells. BORIS expression was induced in two melanoma cell lines (MM057 and MM074) that both harbor a proliferative transcriptional state^4^. Compared to the control cells, we observed a significant increase in the percentage of migrating cells upon BORIS expression (Fig. 4a). Next, we tested the invasive capacity of both cell lines following BORIS expression. In line with the migration data, we observed a significant increase in the percentage of invasive cells for BORpos compared to control cells (Fig. 4b). Collectively, these results demonstrate that BORIS expression enhances the migratory and invasive potential of proliferative melanoma cell lines.

### Identification of putative BORIS target genes among invasion-associated genes

**Fig. 5:**
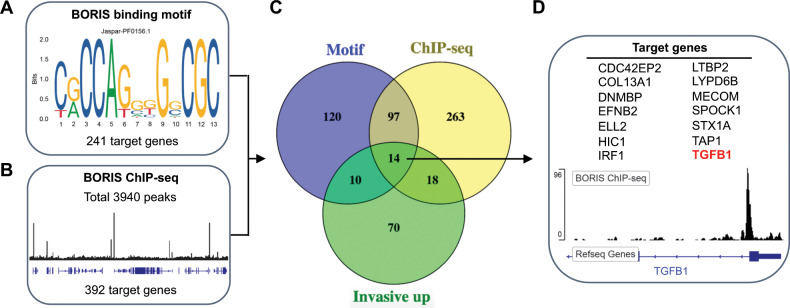
Identification of putative direct BORIS target genes by in silico analysis. **a** iRegulon was used to assess the presence of the depicted BORIS binding motif around the transcription start site of the DEGs (from RNA-seq). Among upregulated DEGs, 241 putative direct BORIS target genes were identified. **b** The BORIS ChIP-seq dataset from ENCODE was provided to the Genomic Regions Enrichment of Annotations Tool, which revealed 3940 BORIS binding sites within 5 kb around the transcription start site. The image represents part of the ENCODE BORIS ChIP-seq track as observed within the Integrative Genomics Viewer. Comparing the genes that contain a BORIS binding site with the upregulated DEGs identified 392 putative direct BORIS target genes. **c** Venn diagram demonstrating the overlap between putative direct BORIS target genes based on the BORIS binding motif (241 genes), BORIS binding site according to the BORIS-ChIP-seq dataset (392 genes), and upregulated DEGs that belong to the invasive gene signatures (112 genes). **d** Overview of the identified 14 putative direct BORIS target genes. The image shows the BORIS binding site in *TGFB1* near the transcription start site based on the ENCODE BORIS ChIP-seq track as observed within the Integrative Genomics Viewer. DEGs differentially expressed genes, ENCODE Encyclopedia of DNA Elements.

To obtain a better understanding of BORIS-mediated transcriptional changes, we used in silico analyses to identify putative direct BORIS target genes among the DEGs from the RNA-seq data. First, we used iRegulon^36^, which identifies master regulators of gene sets based on enrichment for specific DNA-binding motifs around the transcription start site. Motif discovery predicted CTCFL (BORIS) as the most enriched transcription factor-binding motif among the upregulated DEGs. Based on this analysis, 241 putative BORIS target genes were identified (Fig. 5a). Conversely, motif discovery did not reveal enrichment for a BORIS binding motif among downregulated genes. These findings suggest that BORIS may act as a direct transcriptional regulator for a subset of upregulated DEGs.

Next, we used the Genomic Regions Enrichment of Annotations Tool (GREAT)^37^ to extend the list of putative direct BORIS target genes. This tool utilizes cis-regulatory regions obtained from chromatin immunoprecipitation (ChIP)-seq data to identify nearby annotated genes. Publicly available BORIS ChIP-seq data from the ENCyclopedia Of DNA Elements (ENCODE)^38^ was used to identify BORIS binding sites 5 kb around the transcription start site. This analysis revealed 3940 gene promoter regions that were bound by BORIS. From these genes, 392 belonged to the upregulated genes (Fig. 5b) and 96 to the downregulated genes. Consistent with the iRegulon results this analysis revealed a higher number of BORIS binding sites in the promoter of upregulated genes compared to downregulated genes.

Given that BORIS-DNA binding is more prevalent in the promoter region of genes that become transcriptionally activated^23^, we focused on the upregulated DEGs. We compared the upregulated putative BORIS target genes as identified via motif discovery (241 genes) with those identified by ChIP-seq analysis (392 genes), which revealed 111 genes that contain both a BORIS binding motif and were bound by BORIS (Fig. 5c). To elucidate putative direct BORIS target genes among genes of the invasive melanoma gene signatures, we compared these genes to the invasion-associated genes upregulated by BORIS (112 genes). Based on this analysis we identified 14 potential BORIS target genes that are part of the invasive gene signatures (Fig. 5c). Interestingly, among the putative direct BORIS targets we observed TGF-beta (*TGFB1*) (Fig. 5d), which is a well-known inducer of melanoma cell phenotype switching^3,4,39^. These analyses suggest that BORIS can directly modulate the expression of a subset of invasion-associated genes, including *TGFB1*.

### BORIS binds the *TGFB1* gene and upregulates *TGFB1* expression

**Fig. 6:**
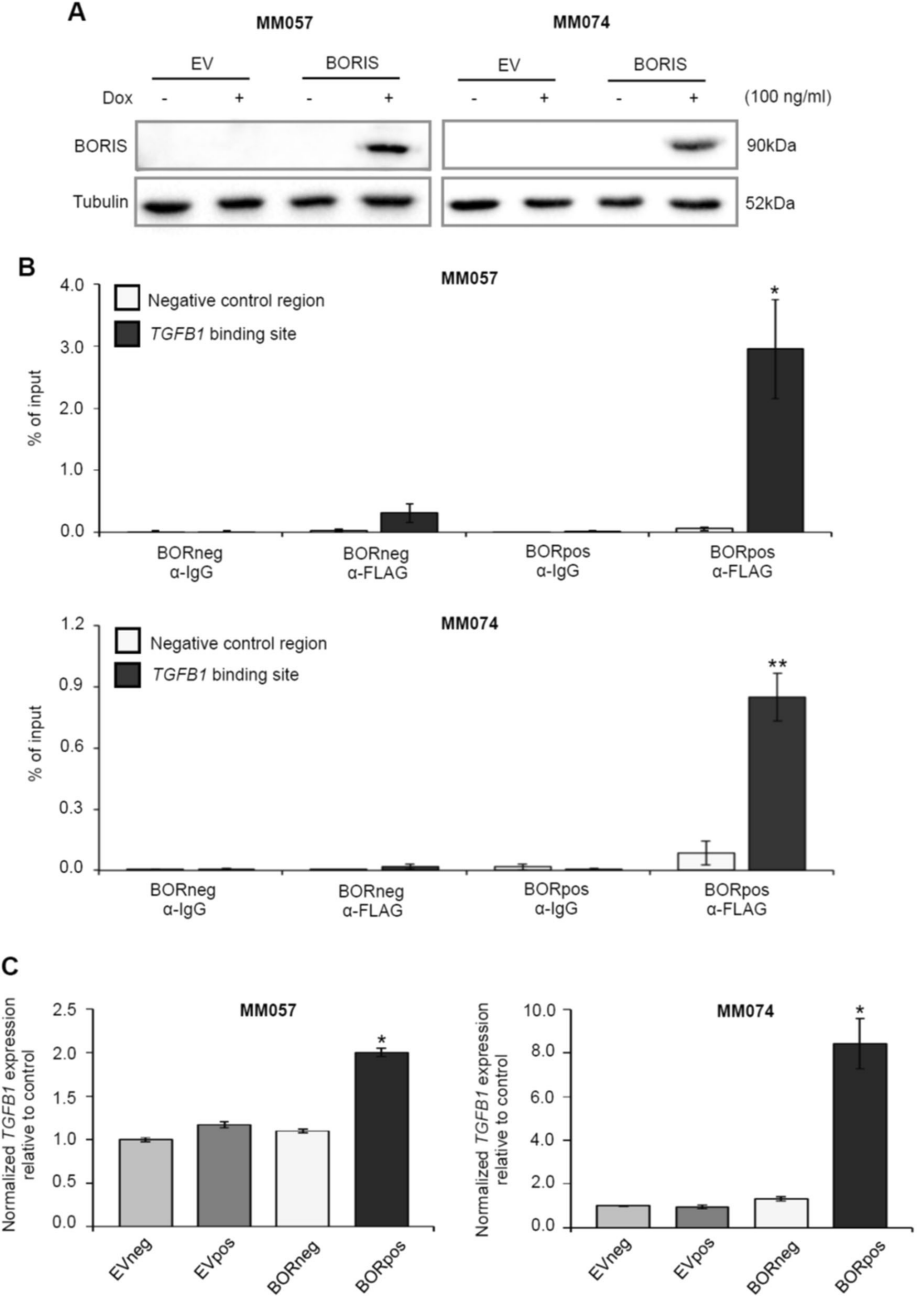
BORIS binds near the *TGFB1* promoter and upregulates *TGFB1* expression in melanoma cells. **a–c** Expression of BORIS fused to a triple FLAG-tag was induced in the MM057 and MM074 melanoma cell lines with 100 ng/ml dox for 5 days. **a** Whole-cell lysate was used for immunoblotting (*n* = 3) with anti-BORIS antibody. Anti-Tubulin was used as a loading control. **b** ChIP with either anti-FLAG antibody or mouse IgG followed by qPCR for either the putative BORIS binding site in *TGFB1* or a negative control region ~3 kbp upstream of the putative binding site. Three independent experiments were performed, each consisting of two technical replicates. **c** qPCR for *TGFB1*. For each qPCR experiment, a technical triplicate was performed for two biological replicates. Expression was normalized to *HPRT1* and *TBP*. Error bars represent the standard error of the mean. Asterisk indicates significantly different from controls (*P < 0.05. **P < 0.01). Dox doxycycline.

Having identified *TGFB1* as a putative direct BORIS target gene, we set out to validate the in silico analysis in melanoma cells. BORIS binding in the *TGFB1* gene was assessed using ChIP followed by qPCR. We generated a dox-inducible expression construct coding for BORIS fused to a triple FLAG-tag, as commercially available BORIS antibodies are not suitable for ChIP. BORIS expression was induced in the MM057 and MM074 cells and expression was confirmed by immunoblot (Fig. 6a). Compared to the controls, we observed a significant enrichment in the immunoprecipitation of BORIS in the *TGFB1* gene in both melanoma cell lines (Fig. 6b), demonstrating that BORIS physically binds the in silico identified binding site.

Next, we assessed if BORIS-DNA binding in *TGFB1* is accompanied by a change in *TGFB1* expression. Consistent with the RNA-seq data, we found increased *TGFB1* levels in BORpos cells compared to the control cells (Fig. 6c), indicating that BORIS binding in the *TGFB1* gene is accompanied by altered *TGFB1* expression. In addition to *TGFB1*, our RNA-seq data revealed increased expression of various genes belonging to the TGF-beta ligand family (Fig. S6A). Furthermore, multiple TGF-beta target genes are significantly upregulated in the RNA-seq dataset, including *FBN1*, *SERPINE1*, *JUNB*, *FOSB*, and *NGFR/CD271* (Figure S6B). Importantly, *CD271*, *SNAI1*, *JUNB*, and *FOSB*, the latter two as part of the AP-1 transcription factor complex, are all known regulators of melanoma phenotype switching^4,8,40^. Together, these data support a direct role for BORIS in the expression of the EMT-inducer TGF-beta and its target genes, revealing one mechanism through which BORIS can modulate the transcriptional state and contributes to an invasive phenotype in melanoma cells.

## Discussion

BORIS is a DNA-binding protein with high similarity to the multifunctional transcription factor CTCF. In contrast to ubiquitously expressed CTCF, BORIS expression is restricted to the testis^12^ and becomes aberrantly reactivated in various types of cancer, including melanoma^41^. In this study, we demonstrate that BORIS can alter the gene expression program of melanoma cells in favor of a more invasive phenotype. Transcriptional profiling revealed that genes upregulated in melanoma cells with ectopic BORIS expression are enriched among established invasive gene signatures, while downregulated genes are enriched among the proliferative gene signatures. In line with these findings, we showed that BORIS expression resulted in reduced proliferation, whereas the migratory and invasive abilities of melanoma cells were enhanced. Furthermore, BORIS’ ability to bind near the *TGFB1* promoter, which coincides with the upregulation of *TGFB1* and TGF-beta target genes, indicates a direct role for BORIS in melanoma phenotype switching. Together, our data show that BORIS contributes to the switch from a proliferative towards an invasive state at both the transcriptional and phenotypic level in melanoma cells.

Recent studies reported BORIS expression in female germ cells and various somatic tissues^41,42^, which challenges the initial description of BORIS as a cancer testis antigen. If BORIS is indeed expressed at the protein level in somatic tissues remains a debate^24^. At the RNA level, *BORIS* is expressed in most normal tissues, though at a much lower level compared to testis. In normal skin we found that *BORIS* expression is very low with detectable levels of *BORIS* in only one third of the samples, while expression in melanoma is significantly higher. Along the same lines, we were unable to detect *BORIS* expression in non-malignant giant nevi cells, while we did measure *BORIS* expression in melanoma cell lines. This is consistent with previous work demonstrating *BORIS* expression in melanoma cell lines and samples, but not in normal skin tissue^33^. Unfortunately, due to the lack of a good commercially available BORIS antibody we were unable to reliably detect endogenous BORIS protein by either immunoblot or immunohistochemistry.

BORIS is involved in numerous cellular processes^41,43^, many of which are altered during carcinogenesis. Here, we used RNA-seq to gain insight into BORIS-regulated cellular processes that contribute to melanoma development and progression. To verify that the transcriptional changes observed in BORpos cells are not a result of BORIS-induced changes in CTCF expression, as observed in other cell types^19,44^, we confirmed a lack of association between BORIS and CTCF expression in both melanoma cell lines and samples. In line with BORIS’ function as a transcriptional regulator^17,18,22,26^, we observed large-scale gene expression changes that indicated a role for BORIS in transcriptional reprogramming of melanoma cells from a proliferative to an invasive state. This process of phenotype switching is critical for melanoma cells to acquire an invasive phenotype that fuels disease progression^45^. In agreement with the acquisition of a pro-invasive transcriptional state, we demonstrated that BORIS-expressing melanoma cells display reduced proliferation and increased migratory and invasive abilities. Reduced proliferation was also observed in previous studies in both primary cells and various cancer cell lines^46,47^, though the literature regarding cell proliferation in the context of BORIS is not uniform^17,20,34^. These discrepancies suggest a cell type specific role for BORIS, which may be mediated by differences in genome-wide BORIS-DNA binding between cell types, as recently suggested^23^. In accordance with our findings, involvement of BORIS in EMT, a process in epithelial cells that is similar to phenotype switching, has previously been observed in different cell types^18,48,49^. Multiple studies found that increased BORIS expression is linked to poor prognosis and more advanced stages of disease, hence BORIS being considered an oncogene^50–53^. Our data indicates a pro-invasive role for BORIS in melanoma and supports the notion that BORIS acts pro-oncogenic.

The large number of genes that belong to the proliferative and invasive gene signatures highlights the complexity that underlies the process of phenotype switching. Multiple transcription factors, signalling pathways and EMT-related genes are important to establish these transitions^3,4,8–11^. Here, we used two complementary in silico approaches and identified putative direct BORIS target genes that could contribute to the BORIS-induced transcriptional switch toward and invasive state. Since the only ENCODE ChIP-seq dataset for BORIS is derived from a myeloid leukemia cell line (K562), we cannot exclude the possibility that the analysis missed certain melanoma-specific BORIS target genes. Nevertheless, our analysis did reveal a larger number of potential direct BORIS target genes. In agreement with the observation that BORIS-DNA binding is enriched at chromatin regions marked by activating histone modifications^23^, the majority of the identified putative direct BORIS target genes were among the upregulated DEGs. We focused our validation experiments of the in silico determined BORIS target genes on *TGFB1*, since TGF-beta driven signaling is an important mark of the invasive signature^1,4^ and TGF-beta acts as tumor promoter at later stages of melanoma tumor development^54^. In this study, we demonstrate the ability of BORIS to bind near the *TGFB1* promoter and show that ectopic BORIS expression leads to increased *TGFB1* levels. In addition, the expression of TGF-beta family members and TGF-beta target genes was increased, which is likely mediated by autocrine and paracrine signaling between tumor cells. Importantly, among the TGF-beta target genes that were upregulated in our RNA-seq dataset are *CD271*, *SNAI1*, and *JUNB*, which are all known regulators of melanoma phenotype switching^4,8,40^. Notably, a previous study that characterized *Ctcfl* transgenic mice observed a phenotype that resembled mice with an altered TGF-beta pathway. RNA-seq of the *Ctcfl* transgenic embryonic stem cells revealed disruption of the TGF-beta pathway as well as increased *TgfB1* expression^55^, which is in line with our observations. These observations support the idea that BORIS acts as a transcriptional regulator of phenotype switching via *TGFB1* and warrant further investigations into the role of BORIS in TGF-beta signaling.

Herein we report that BORIS expression induces phenotype switching, at least in part via upregulation of TGF-beta signaling. Other mechanisms may contribute to BORIS-mediated transcriptional changes, including competition with CTCF for DNA-binding sites, epigenetic modifications, and/or chromatin looping, which are the subject of further investigations. Taken together, our study demonstrates that BORIS contributes to melanoma progression by intrinsically rewiring gene expression to promote an invasive phenotype. Furthermore, these results indicate that BORIS is an important transcriptional regulator during phenotype switching in melanoma and provide a rationale for further studies into BORIS’ role as an invasion promoting transcriptional regulator.

## Materials and methods

### Cell lines

Early passage melanoma cell lines (MM102, MM117, MM073, MM057, MM079, and MM074) and giant congenital nevi cell lines (GCN004, GCN008, and GCN011) were a kind gift from Dr. G. Ghanem (Institut Jules Bordet, Brussels, Belgium) and were cultured in Ham’s F10 medium. The SK-MEL-2, SK-MEL-5, MALME-3M, WM164 and WM983B cell lines were purchased from ATCC and cultured in RPMI 1640 medium. HEK293T cells were purchased from ATCC and cultured in DMEM. For all cell lines the medium was supplemented with 8% heat-inactivated fetal bovine serum, 1% penicillin-streptomycin and 1% GlutaPlus. Inducible cell lines were selected by the addition of G418 (Neomycin; MM057 150 μg/ml; MM074 200 μg/ml) for a minimum of 2 weeks or until non-infected control cells were killed. Upon selection, MM057 and MM074 cell lines were maintained in the presence of 50 μg/ml G418. Doxycycline (dox; Clontech) was added to the medium for the indicated dose and time, and refreshed every 48 h. Bimonthly tests for mycoplasma demonstrated the absence of contamination. All cultures were maintained at 37 °C in a 5% CO_2_ humidified atmosphere.

### Generation of expression vectors

*pI20-BORIS-6xH*. Full-length human BORIS cDNA was amplified by PCR on RNA extracted from HEK293T cells using iProof (Bio-Rad) with primers containing BamHI and MluI restriction sites (forward 5′-CAGCGGATCCACTGAGATCTCTGTCCTTTCTGAG-3′ and reverse 5′-GCGGACGCGTCT CACTTATCCATCGTGTTGAGGAGCATTTCACAGG-3′). The PCR product was digested with BamHI and MluI (Fermentas FastDigest) and inserted into pDONR221 (Invitrogen cat # 12536017) by a BP reaction (Gateway^TM^ BP Clonase^TM^ II Enzyme mix, Invitrogen) to generate pENTR-BORIS with six C-terminal His-tags followed by a stop codon. To generate pI20-BORIS-6xH the entry clone was recombined into the lentiviral destination vector pI20 (empty vector (EV)-6xH, a gift from Stephen Elledge^56^, Addgene plasmid #44012) using Gateway LR Clonase^TM^ II (Fisher).

*pI20-BORIS-3xF*. Full-length human BORIS cDNA tagged at its C-terminal with three FLAG^®^-tags was amplified by PCR using iProof (Bio-Rad) and *attB* sites were introduced with the following primers: Forward 5′-GGGGACAAGTTTGTACAAAAAAGCAGGCTCCACCATGGCAGCCACTGAGATCT CTGTCC-3′ and reverse 5′- GGGGACCACTTTGTACAAGAAAGCTGGGTCTCACTAGTCACGCTA GCATCTGACGCGCTA-3′. The resulting PCR product was inserted into pDONR221 (Invitrogen) by a BP reaction (Gateway^TM^ BP Clonase^TM^ II Enzyme mix, Invitrogen) to generate pENTR-BORIS-3xF. A pENTR-Fluc-3xF (expressing Firefly luciferase tagged at its C-terminal with three FLAG^®^-tags) was obtained in a similar way. Next, the entry clones were recombined into the pI20 vector using Gateway LR Clonase^TM^ II (Fisher) to generate pI20-BORIS-3xF and the control pI20-Fluc-3xF.

### Transfection, lentivirus production, and infection

For virus production, lentiviral expression vectors (10 μg DNA) were transfected into HEK293T cells along with the viral packaging plasmids pSPAX2 (7.5 μg DNA) and pMD2.G (5 μg DNA) using polyethylenimine (PEI; Polysciences, DNA:PEI ratio of 1:2.5) and Opti-MEM transfection medium (Invitrogen). The medium was replaced by fresh medium 3 h after transfection. At 48 h post transfection, virus-containing supernatant was harvested, centrifuged, passed through a 0.45 μm syringe filter, and either used directly for infection or stored at −80 °C. Cells were infected twice by incubation with a mixture of viral supernatant, medium (1:1 ratio) and polybrene (8 µg/ml, Sigma–Aldrich) for 12 h. The viral mixture was replaced by fresh medium for 24 h before selection. Expression of the constructs was verified using a combination of immunoblot and quantitative PCR (qPCR).

### RNA extraction, reverse transcription, and qPCR

Total RNA was extracted using the Nucleospin RNA kit (Macherey Nagel) according to manufacturer’s instructions. DNase treatment excluded genomic DNA. RNA quantity was determined by NanoDrop. Total RNA (400 ng) was reverse transcribed using the qScript cDNA synthesis kit (Quanta) according to manufacturer’s specifications. qPCR was performed with PerfeCTa SYBR Green FastMix (Quanta) on a CFX96 Touch^TM^ (Bio-Rad). Each 10 μl reaction contained 4 μl cDNA (1/5 diluted), 5 μl PCR mix and 400 nM forward and reverse primer. The thermal cycling program used was as follows: 30 sec at 95 °C followed by 40 cycles of 5 sec at 95 °C and 20 sec at 60 °C, followed by 10 sec at 95 °C and a melt curve with incremental steps of 0.5 °C for 5 sec starting at 65 °C and ending at 95 °C. Primers were designed to be exon spanning when possible. For each primer pair, the melting temperature, GC content and the secondary structure of the PCR product were assessed using tools available from the University of California Santa Cruz Genome Browser (https://genome.ucsc.edu/cgi-bin/hgPcr) and Integrated DNA Technologies (https://www.idtdna.com). Melting curve analyses were carried out to ensure product specificity. Primers were purchased from Integrated DNA Technologies and sequences are provided in Table S1. Importantly, *BORIS* primers span exon 9 and 10, and were designed to amplify isoform family’s sf1 and sf5^57^. Samples were run in technical triplicate and biological duplicate, and data were normalized to both *HPRT1* and *TBP*. All data were analyzed using the CFX ManagerTM Software (Bio-Rad).

### Immunoblot

Cells were grown to 70% confluency and harvested in ice cold PBS by scraping. The collected cell pellets were lysed on ice with lysis buffer (50 mM HEPES-KOH pH7.4, 150 mM NaCl, 1% triton X-100, 0.2% sodium deoxycholate, 5 mM EGTA, 10% glycerol) supplemented with 1x protease inhibitor cocktail (Promega) and phosphatase inhibitor (Roche), and sonicated three times for 15 sec at 25% power. Lysates were cleared by centrifugation and protein concentration was determined using the Pierce BCA protein assay kit (Fisher) according to manufacturer’s instructions. Lysates were denatured in SDS sample buffer for 5 min at 95 °C. For direct lysis, 2 × 10^5^ cells were lysed in 1x SDS sample buffer and denatured for 10 min at 95 °C. Protein extracts were separated on SDS-polyacrylamide gel and transferred onto polyvinylidene difluoride membranes (Bio-Rad). Membranes were blocked with 3% nonfat milk (Bio-Rad) or 3% bovine serum albumin (Bioshop) in PBS containing 0.05% Tween 20 (TBST) and probed with the appropriate primary antibody overnight at 4 °C. Blots were washed with TBST and probed with the corresponding horseradish peroxidase-conjugated secondary antibody. Proteins were detected using enhanced chemiluminescence reagent (Bio-Rad), images were captured with a ChemiDoc^TM^ Imaging System (Bio-Rad) and analyzed with Image Lab^TM^ Software (Bio-Rad Laboratories, Version 5.0 build 18).

The following antibodies were used: rabbit anti-human BORIS (Rockland, 600-401-907), rabbit anti-human CTCF (Millipore, 07–729), rabbit anti-human PARP (Cell Signaling, 9542), and rabbit anti-human cleaved PARP (Cell signaling, 5625) at a 1:1000 dilution, and rat anti-human tubulin (Abcam, ab6160) at 1:7000. Secondary antibodies used were goat anti-rabbit (Jackson, 115-035-144) and anti-rat IgG (Cell Signaling, 7077) coupled to horseradish peroxidase.

### Proliferation

Cell proliferation was assessed by crystal violet assay as described by Krayem et al.^58^. Based on the number of days that the cells would be cultured to assess proliferation, cells were seeded in a 96-well plate at either 5 × 10^3^ cells (for 3 days), 4 × 10^3^ cell (for 5 days), or 2 × 10^3^ cells (for 7 days) per well. The following day, cells were treated with dox (Clontech) for the indicated concentration and after either 3, 5, or 7 days in the presence of dox, cells were fixed using 4% formaldehyde (Fisher). Next, cells were stained with 0.05% crystal violet (Sigma–Aldrich) and washed three times with ddH2O to remove excess crystal violet. Once the plates dried, crystal violet was solubilized overnight using 150 μl 0.2% Triton X-100 (Bioshop) and absorbance was measured using the FLUOstar OPTIMA (BMG Labtech) at 570 nm. Results are displayed as percentage of cells compared to untreated. Two independent experiments were performed in technical triplicate.

### Apoptosis assay

Apoptosis was assessed by AnnexinV/PI staining. Cells (2 × 10^5^) were seeded in a 6-well plate and treated with or without 50 ng/ml dox the next day. After 5 days, cells and their supernatant were collected and washed with cold PBS. Cells were stained for AnnexinV and PI by incubation in 100 μl staining solution (1 μl APC-conjugated AnnexinV (eBioscience), 0.1 μg PI (Invitrogen), 1× binding buffer) for 15 min at room temperature in the dark. Next, cells were resuspended in 1× binding buffer and results were immediately acquired on a FACSCalibur^TM^ flow cytometer (BD Biosciences) and analyzed using *FlowJo*^®^ software (version 10.4.1). Three independent experiments were performed.

### Migration and invasion

In vitro cell migration and invasion was evaluated using transwell assays. Cells were treated with or without 100 ng/ml dox for five days, counted and seeded at 5 × 10^4^ (MM057) or 1 × 10^5^ (MM074) cells in serum-free medium in the upper transwell insert chamber (PET membrane, 12-well, 8-μm pores; Corning Costar). For the invasion assays, the insert chamber was coated with Matrigel® (Corning) diluted 1 in 5 with cold, serum-free medium. Complete medium (900 μl) with 8% FBS was added to the lower chamber and cells were allowed to migrate and invade for either 16 h (MM057) or 72 h (MM074) at 37 °C in a 5% CO_2_ humidified atmosphere. The cells that did not migrate were removed from the top of the transwell membrane using a cotton swab. Migrated cells were fixed with 3.7% paraformaldehyde, permeabilized in methanol and stained with crystal violet (0.5%). Inserts were washed with ddH2O to remove excess crystal violet. Images were captured of six fields per insert chamber using the EVOS FL Cell Imaging System (ThermoFisher Scientific). Cells were counted with the Cell Counter option in ImageJ. Representative images were captured using an inverted light microscope (Olympus BX43). Three independent experiments were performed, each consisting of two technical replicates.

### Chromatin immunoprecipitation (ChIP) followed by qPCR

Two 15-cm dishes of cells were treated with or without 100 ng/ml dox for five days, grown to ~70% confluence and crosslinked with 1% methanol-free formaldehyde for 7 min at room temperature. The reaction was quenched using 0.125 M glycine/PBS for 10 min at room temperature. Cells were washed, harvested in cold PBS supplemented with 1× protease inhibitor cocktail (Promega), and pelleted by centrifugation. Cell pellets were resuspended in cold hypotonic buffer (10 mM Tris-HCl pH7.4, 10 mM NaCl, 3 mM MgCl_2_, 0.5% Igepal CA-360) and placed on ice for 15 min. Nuclei were obtained by centrifugation at 3500 rpm for 5 min at 4 °C, then washed with cold hypotonic buffer and stored at −80 °C until further use. The nuclear pellets were resuspended in shearing buffer (50 mM Tris-HCl pH 8.0, 1 mM EDTA, 1% Triton X-100, 0.3% SDS, 0.3% Na deoxycholate) supplemented with 1× protease inhibitor cocktail (Promega). Chromatin was sheared using an Adaptive Focused Acoustics^TM^ ultrasonicator (Covaris) with the following settings: Intensity Peak: 75 watts, Duty cycle: 20%, Cycles per burst: 600 and Processing time: 900 sec. Sheared samples were transferred to a pre-chilled microcentrifuge tube containing dilution buffer (5 mM Tris-HCl pH 8.0, 1 mM EDTA, 210 mM NaCl, 1% Triton X-100) and ChIP buffer (20 mM Tris-HCl pH 8.0, 1 mM EDTA, 150 mM NaCl, 1% Triton X-100, 0.1% SDS, 0.1% sodium deoxycholate) supplemented with 1× protease inhibitor cocktail (Promega), and centrifuged at 13,000 rpm for 5 min at 4 °C. From each sample, 1% (5 μl) was kept as input and 0.5 ml was combined with Dynabeads Protein G conjugated with either 5 μg mouse anti-FLAG M2 antibody (Sigma, F1804, clone M2) or mouse IgG (Sigma, I5381). Mixtures were incubated on a rotator overnight at 4 °C. The ChIP’ed immune complexes were successively washed four times with Low Salt Buffer (20 mM Tris-HCl pH 8.0, 2 mM EDTA, 150 mM NaCl, 1% Triton X-100, 0.1% SDS), High Salt Buffer (20 mM Tris-HCl pH 8.0, 2 mM EDTA, 500 mM NaCl, 1% Triton X-100, 0.1% SDS) and LiCl Buffer (20 mM Tris-HCl pH 8.0, 1 mM EDTA, 0.25 M LiCl, 0.5% IGEPAL CA-630, 0.5% Na deoxycholate) and decrosslinked with 4 μg/ml proteinase K overnight at 65 °C. Samples were treated with 2 μg/ml RNase A at 37 °C for 30 min followed by double size selection with AMPure XP beads (0.6× and 1.2×, Beckman Coulter). DNA was eluted with ddH_2_O and used for qPCR on a CFX96 Touch^TM^ (Bio-Rad). Each 10 μl reaction contained 2.5 μl template, 5 μl PerfeCTa SYBR Green FastMix (Quanta) and 200 nM forward and reverse primer. Specific primers were designed for the putative BORIS binding site near the *TGFB1* promoter, as obtained from the Encyclopedia of DNA Elements (ENCODE) CTCFL ChIP-seq track (ENCSR000BNK), and ~3 kbp upstream of the putative binding site (negative control). Primers were purchased from Integrated DNA Technologies and sequences are provided in Table S1. The thermal cycling program used was as follows: 5 min at 95 °C followed by 40 cycles of 15 sec at 95 °C and 20 sec at 60.5 °C, followed by 10 sec at 95 °C and a melt curve with incremental steps of 0.5 °C for 5 sec starting at 65 °C and ending at 95 °C. Primer efficiencies were determined by standard curve analysis and incorporated in the delta-Ct method to calculate percent enrichment compared to input. Three independent experiments were performed, each consisting of two technical replicates.

### RNA-sequencing

Total RNA was extracted using the Nucleospin RNA kit (Macherey Nagel) according to manufacturer’s instructions. DNase treatment excluded genomic DNA. Total RNA of three biological replicates for each experimental condition was submitted to the Centre for Applied Genomics in Toronto (ON, Canada) for RNA quantitation and quality assessment, library preparation and sequencing. RNA quality and quantity were assessed on a Bioanalyzer (Agilent Technologies). RNA integrity numbers >8.9 were obtained. RNA-seq libraries were prepared using the NEBNext Poly(A) mRNA Magnetic Isolation Module (New England Biolabs) to enrich for poly(A) mRNA and the NEBNext Ultra Directional RNA Library Prep Kit for Illumina (New England Biolabs), according to manufacturers’ specifications. Libraries were indexed using the NEBNext Multiplex Oligos 1–8 for Illumina (Index Primers Set 1; New England Biolabs), according to manufacturers’ specifications. Eight samples per lane were sequenced on a HiSeq2500, using a high throughput V4 flowcell to generate paired-end reads of 126-bases.

### RNA-sequencing analysis

FASTQ files were aligned to the human reference genome GRCh38.p12 using the STAR RNA-seq aligner^59^ with default parameters. BAM files were indexed and name sorted with SAMtools^60^. Further downstream analyses, such as RNA transcripts quantification and analysis of differentially expressed genes (DEGs), were conducted as in ref. ^61^ The HTSeq framwork^62^ was used to count the number of reads that mapped to each gene, with the following command; python -m HTSeq.scripts.count -f bam -r name -s no -i gene_name gencode.v28.annotation.gtf. The CPM cutoff was set at 0.5 CPM (13.5 reads on average, range: 12–14.5 reads) in all three biological replicates of at least one treatment condition. In order to identify DEGs, the Bioconductor edgeR^63^ gene-wise exact-test was used. A total of 11 DEGs were identified between EVneg and EVpos (with a relaxed statistical cutoff, using a Benjamini and Hochberg^64^ false discovery rate (FDR) cutoff of 0.5) and removed from further analyses (Table S2). Next, with FDR < 0.05 and a |log_2_ fold-change (log_2_FC)| > 0.5, a total of 2045 DEGs were identified between BORneg and BORpos (737 downregulated and 1308 upregulated; including *BORIS*; Table S3).

### Gene set enrichment analysis

GSEA (version 3.0 build 0160)^35^ was used on the RNA-seq data for enrichment analysis. The RNA-seq recommended GSEA-PreRanked algorithm was used with 10^4^ permutations, ranking genes by log_2_FC values (as obtained from the edgeR analysis of BORneg vs BORpos samples). The GO gene set for biological processes and the Hallmark epithelial-mesenchymal transition gene set (M5930 version 5.0) from the Molecular Signatures Database^65^ were used. In addition, two proliferative and two invasive published melanoma gene sets were used^3,4^. To determine the overlap between upregulated or downregultated DEGs and genes within the signatures, genes from the Hoek and Verfaillie invasive/proliferative signatures were combined. The hypergeometric test (phyper(q, m, n, k, lower.tail = TRUE, log.p = FALSE)) was used in an online R environment (R v3.4.1) to calculate the probability of gene list overlap.

### iRegulon

Transcriptional regulator prediction was performed using the iRegulon plugin (version 1.3)^36^ in Cytoscape (version 3.6.1. Java version: 1.8.0_171)^66^. The list of either BORIS upregulated or downregulated DEGs was used as input and analyzed using motif collection on a 20-kb region centered around the transcription start site for seven species, which included 9713 position weight matrices. The target genes provided with the BORIS binding motif were used for downstream analysis.

### Genomic regions enrichment of annotations tool

The UCSC table browser was used to submit the ENCODE CTCFL ChIP-seq track BED file (experiment ENCSR000BNK, file ENCFF472SHK^38^) as output to the Genomic Regions Enrichment of Annotations Tool (GREAT)^37^. The associated genomic regions parameter was set as follows: Basal plus extension, proximal 2.5 kb upstream, 2.5 kb downstream, plus distal up to 5.0 kb. The gene-genomic region association table was downloaded for downstream analysis.

### Analysis of publicly available datasets

*BORIS* expression data for normal skin and testis (Genotype-Tissue Expression (GTEx)), and melanoma samples (The Cancer Genome Atlas (TCGA), Skin Cutaneous Melanoma dataset (SKCM)) was downloaded from the TCGA TARGET GTEx cohort through the UCSC Xena platform^67^. This provides the same expression unit between the GTEx and TCGA databases (file: rsem.genes.norm_counts, log_2_(x + 1) transformed expression values). Expression differences between datasets were analyzed using Welch’s *t*-test. *BORIS* and *CTCF* expression data for primary and metastatic melanoma samples were obtained from the GDC TCGA melanoma cohort through the UCSC Xena platform^67^ (expression unit: log_2_(x + 1) transformed FPKM-UQ). The excel StatPlus:mac analysis package (build 6.8.0.0/Core v6.9.94) was used to perform multiple linear regression analysis between *BORIS* and *CTCF* expression.

### Statistical analyses

The numbers of biological and technical replicates are specified in the method section for each experimental procedure. Except for the analysis of the RNA-seq, public dataset, and GSEA, all statistical evaluations were carried out by unpaired, two-tailed Student’s *t*-test. *P* < 0.05 was considered statistically significant. Data are represented as mean ± standard error of the mean.

## Supplementary information

Supplementary figures and Legends

Supplementary Table I-111 and Legends

Table S1-qPCR primers

Table S2-DEGs EVneg vs EVpos

Table S3-DEGs BORneg vs BORpos

## Data Availability

The RNA-seq data from this publication have been deposited to the Gene Expression Omnibus (GEO; https://www.ncbi.nlm.nih.gov/geo/) and assigned the GEO accession number GSE126807.
