# Supplementary material for: BORIS/CTCFL promotes a switch from a proliferative towards an invasive phenotype in melanoma cells
Source: Cell Death Discov. 2020 Jan 2;6:1. doi: 10.1038/s41420-019-0235-x (PMC7026120; doi:10.1038/s41420-019-0235-x)
Supplement: Supplementary file 1 — Supplementary figures and Legends [file 41420_2019_235_MOESM1_ESM.docx]

**
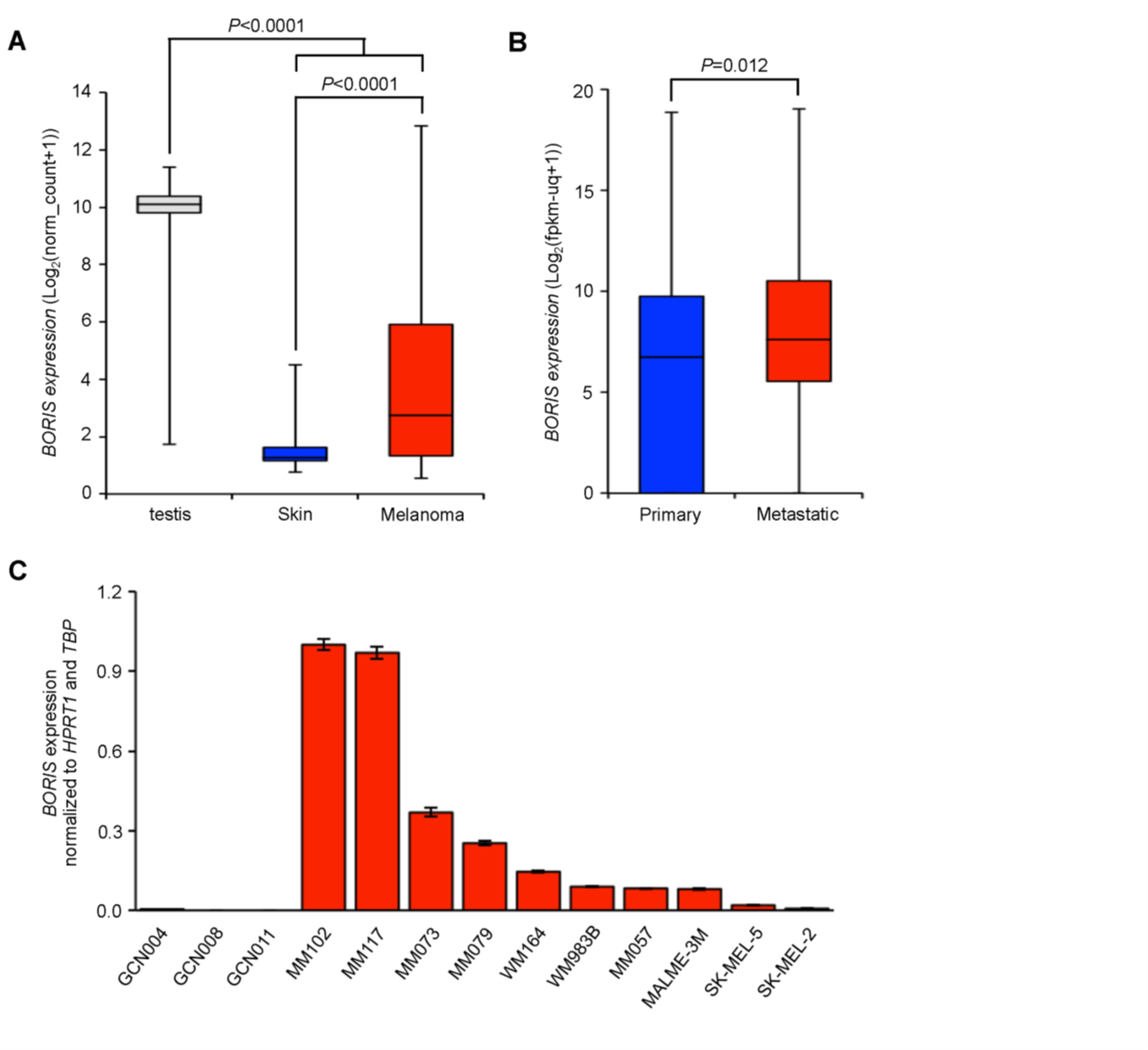
**

**Figure S1. *BORIS* expression in skin, melanoma samples, and melanoma cell lines.**

**(A, B)** Boxplot visualizing *BORIS* expression obtained through the UCSC Xena platform data for **(A)** testis, skin and melanoma samples, and **(B)** primary and metastatic melanoma samples. **(C)** Relative *BORIS* expression as determined by qPCR. A technical triplicate was performed for two biological replicates. Expression was normalized to *HPRT1* and *TBP*. Error bars represent the standard error of the mean. fpkm: fragments per kilobase per million, uq: upper quartile.

**
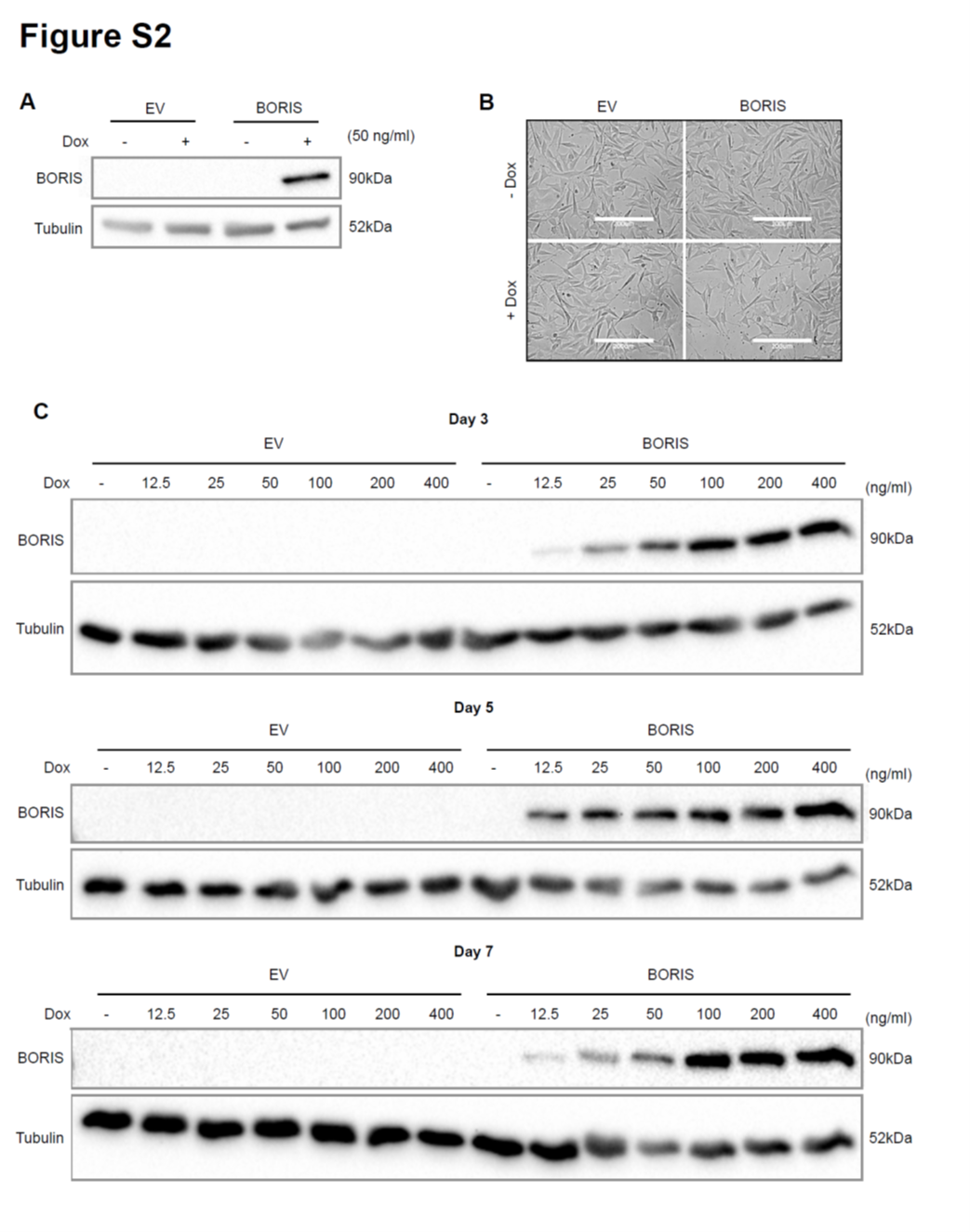
**

**Figure S2. Doxycycline-inducible dose-dependent BORIS overexpression and reduced proliferation in melanoma cells.**

**(A, B)** Expression of BORIS was induced in the MM057 cell line with 50ng/ml dox for 5 days. **(A)** Whole cell lysate was used for immunoblotting with anti-BORIS and anti-Tubulin antibodies (image used in Figure 1D). **(B)** Bright-field image during culture of control cells and cells with ectopic BORIS expression. **(C)** Expression of BORIS was induced with the indicated concentration of dox for 3, 5 or 7 days of cell culture. Whole cell lysate was used for immunoblotting with anti-BORIS antibody. Anti-Tubulin was used as a loading control. Dox: doxycycline, EV: empty vector.


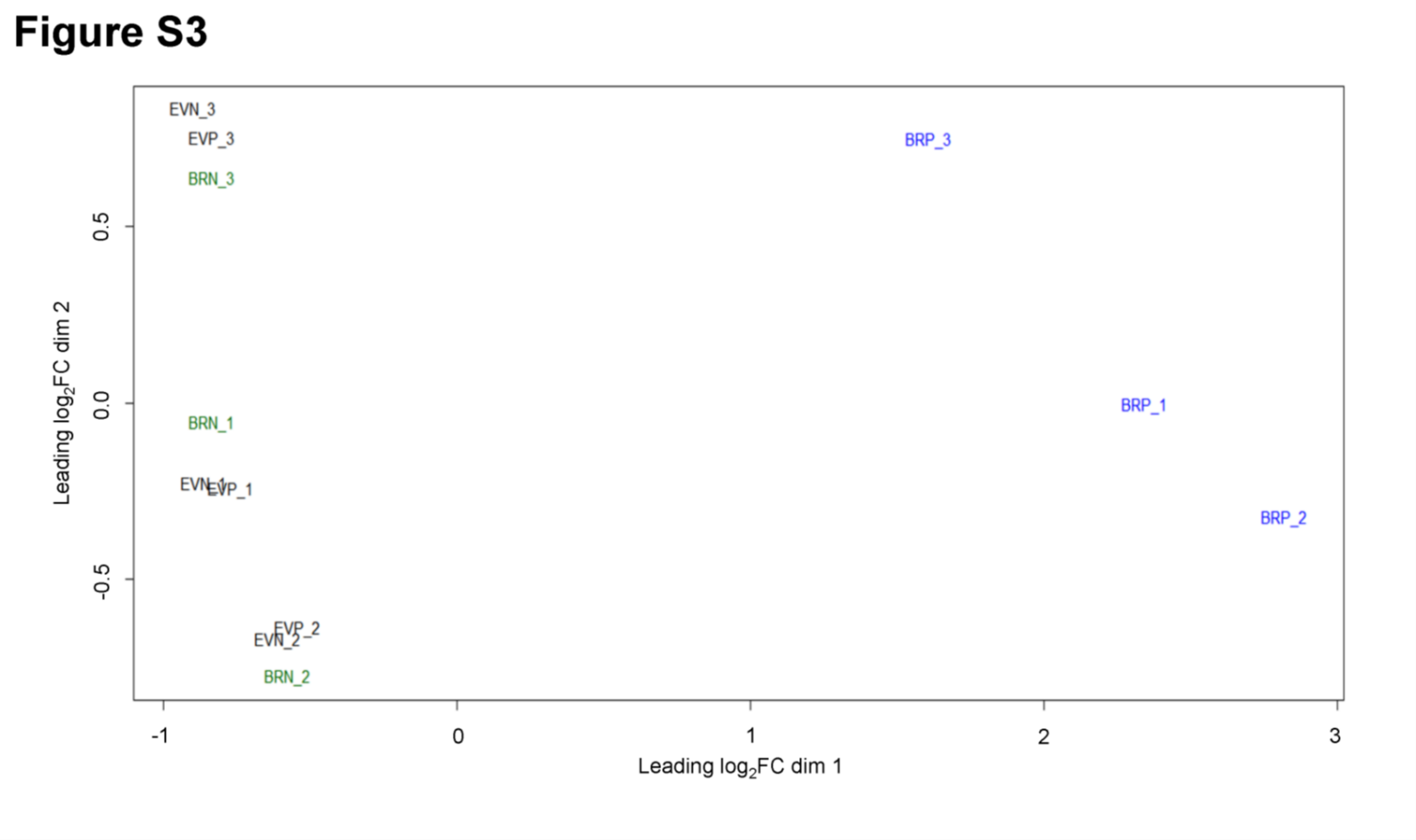


**Figure S3. Clustering of RNA-seq control samples versus samples with ectopic BORIS expression**

Multidimensional scaling plot to visualize the level of similarity between each RNA-seq sample. EVN: EVneg, EVP: EVpos, BRN: BORneg, BRP: BORpos, dim 1: dimension 2, dim 2: dimension 2, log_2_FC: log_2_ fold change.

**
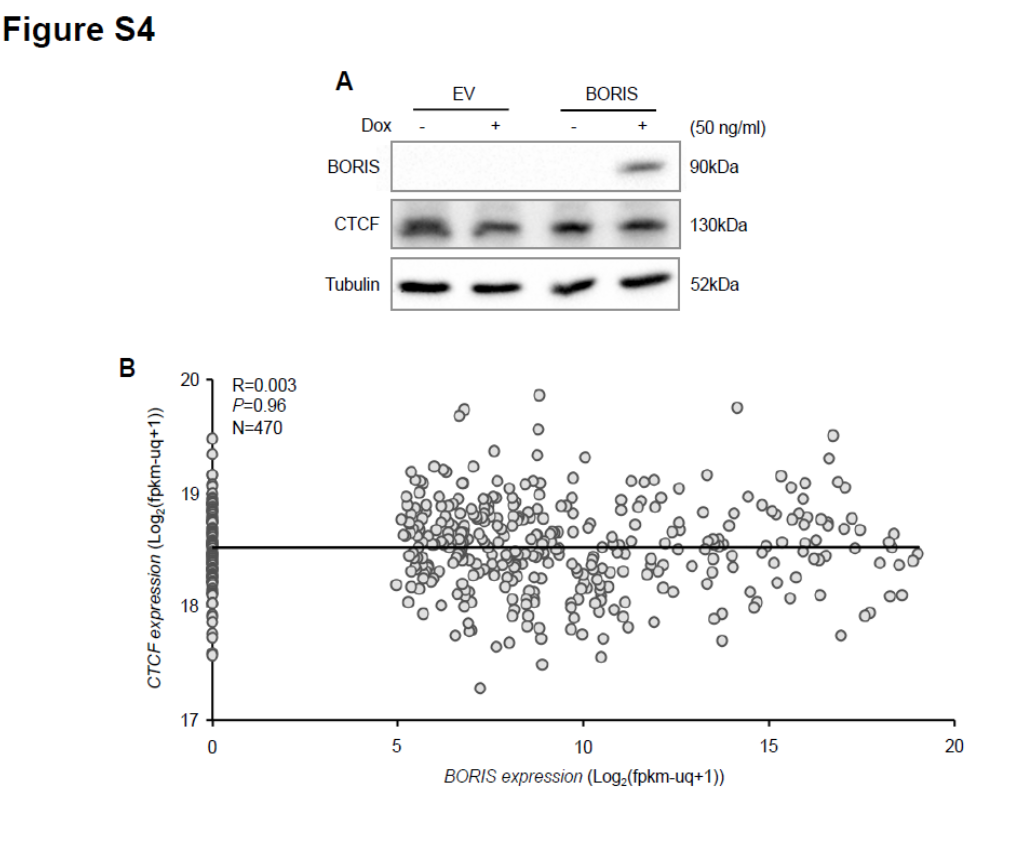
**

**Figure S4. No correlation between BORIS and CTCF expression in melanoma.**

**(A)** Expression of BORIS was induced in the MM057 cell line with 50ng/ml dox for 5 days. Whole cell lysate was used for immunoblotting with anti-BORIS or anti-CTCF antibodies. Anti-Tubulin was used as a loading control. **(B)** *BORIS* and *CTCF* expression in melanoma samples from the TCGA SKCM dataset. Dox: doxycycline, R: correlation coefficient, N: number of samples, fpkm: fragments per kilobase per million, uq: upper quartile.

**
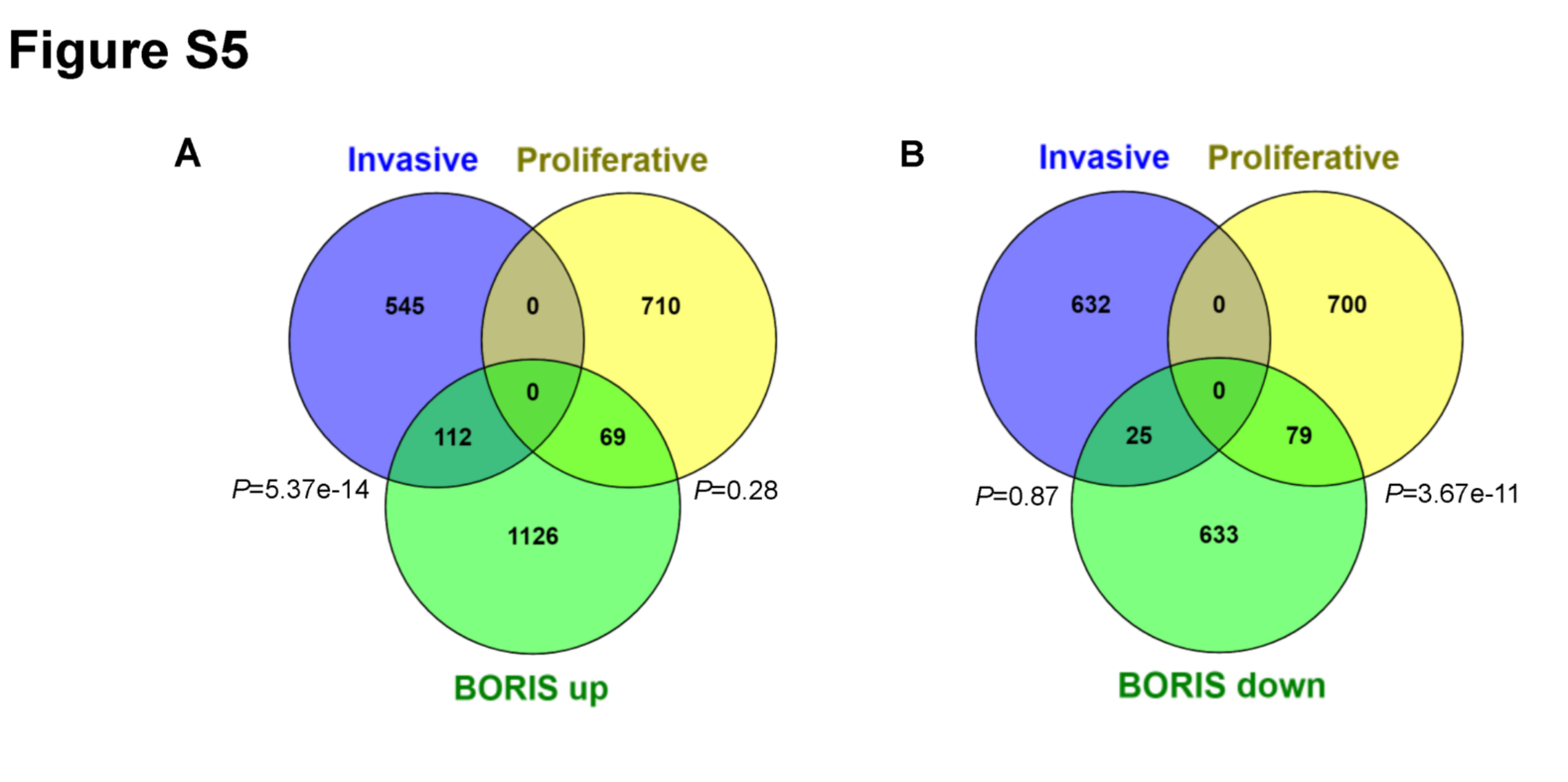
**

**Figure S5. Upregulated DEGs overlap with the invasive signatures, while downregulated DEGs overlap with the proliferative signatures.**

Venn diagram demonstrating the overlap between genes from the invasive or proliferative signature and either **(A)** upregualted DEGs or **(B)** downregulated DEGs.

**
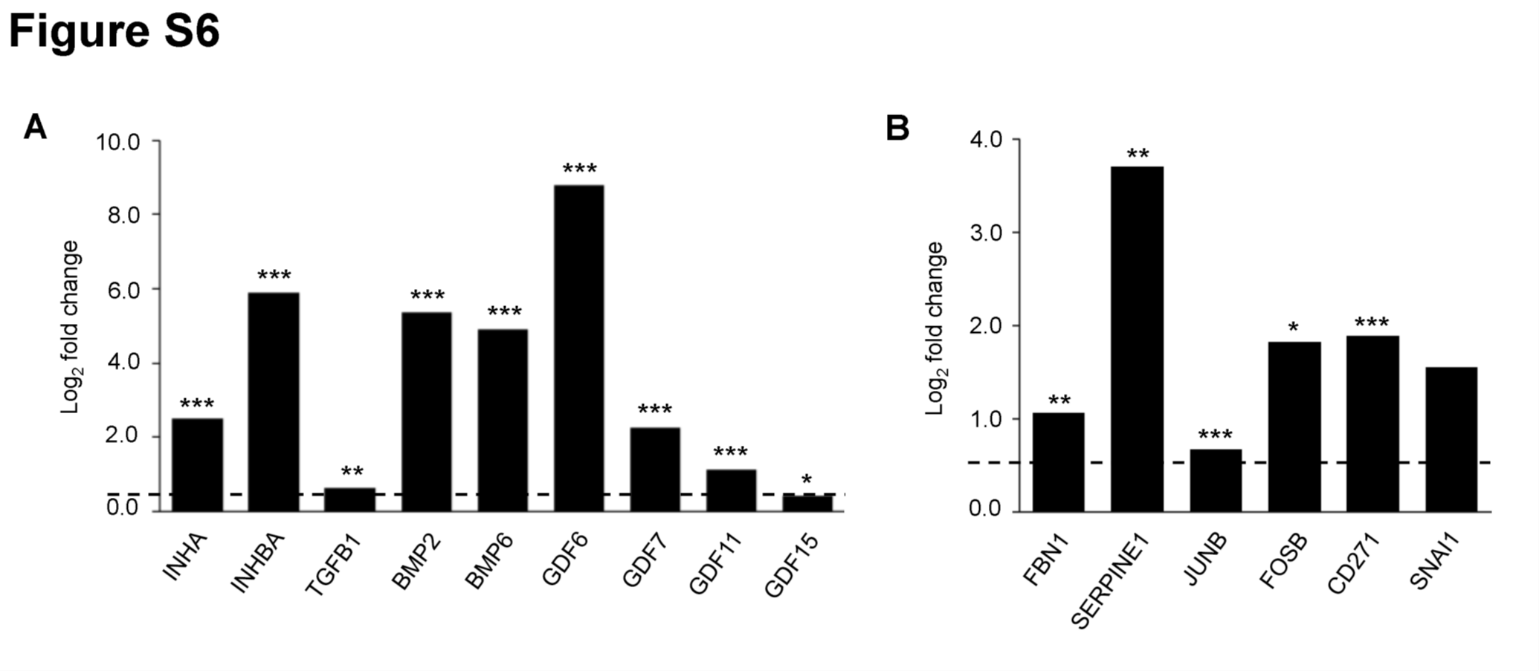
**

**Figure S6. BORIS expression leads to increased expression of TGF-beta family members and TGF-beta target genes.**

**(A, B)** Bar graph representing the log_2_ fold change between BORneg and BORpos RNA-seq samples.
