# Supplementary material for: BORIS/CTCFL promotes a switch from a proliferative towards an invasive phenotype in melanoma cells
Source: Cell Death Discov. 2020 Jan 2;6:1. doi: 10.1038/s41420-019-0235-x (PMC7026120; doi:10.1038/s41420-019-0235-x)
Supplement: Supplementary file 2 — Supplementary Table I-111 and Legends [file 41420_2019_235_MOESM2_ESM.docx]

**Supplementary Table Legends**

**Table S1. qPCR and ChIP primers**

This table contains the nucleotide sequence of the primers used for qPCR and ChIP-qPCR.

**Table S2. DEGs EVneg vs EVpos**

This table contains the results of the edgeR gene-wise exact-test between the EVneg and EVpos RNA-seq samples (3 biological replicates). logFC: log_2_ fold change, logCPM: log_2_ counts per million; FDR: false discovery rate.

**Table S3. DEGs BORneg vs BORpos**

This table contains the results of the edgeR gene-wise exact-test between the BORneg and BORpos RNA-seq samples (3 biological replicates). logFC: log_2_ fold change, logCPM: log_2_ counts per million; FDR: false discovery rate.
